# Tuberculosis Outbreak Investigations in the United States, 2002–2008

**DOI:** 10.3201/eid1703.101550

**Published:** 2011-03

**Authors:** Kiren Mitruka, John E. Oeltmann, Kashef Ijaz, Maryam B. Haddad

**Affiliations:** Author affiliation: Centers for Disease Control and Prevention, Atlanta, Georgia, USA

**Keywords:** Bacteria, tuberculosis and other mycobacteria, TB, Mycobacterium tuberculosis, disease outbreaks, public health, outbreak investigation, United States, substance abuse, research

## Abstract

To understand circumstances of tuberculosis transmission that strain public health resources, we systematically reviewed Centers for Disease Control and Prevention (CDC) staff reports of US outbreaks in which CDC participated during 2002–2008 that involved >3 culture-confirmed tuberculosis cases linked by genotype and epidemiology. Twenty-seven outbreaks, representing 398 patients, were reviewed. Twenty-four of the 27 outbreaks involved primarily US-born patients; substance abuse was another predominant feature of outbreaks. Prolonged infectiousness because of provider- and patient-related factors was common. In 17 outbreaks, a drug house was a notable contributing factor. The most frequently documented intervention to control the outbreak was prioritizing contacts according to risk for infection and disease progression to ensure that the highest risk contacts were completely evaluated. US-born persons with reported substance abuse most strongly characterized the tuberculosis outbreaks in this review. Substance abuse remains one of the greatest challenges to controlling tuberculosis transmission in the United States.

Among the major challenges in achieving tuberculosis (TB) elimination in the United States are preventing, detecting, and responding to TB outbreaks. Identifying high-risk settings and applying effective control measures to reduce TB transmission are basic principles of TB control. Since the 1985–1992 TB resurgence in the United States, enhanced infection control measures in health care facilities have successfully reduced nosocomial transmission and outbreaks (*1*). However, outbreaks in community settings have continued to occur, calling for increased vigilance in understanding and controlling TB transmission (*1*).

When health departments determine that they have exceeded their surge capacity to control a TB outbreak, the Centers for Disease Control and Prevention (CDC) Division of Tuberculosis Elimination may be invited to assist. During an onsite investigation lasting ≈2–3 weeks, CDC works closely with its public health partners to describe the epidemiology of the outbreak, find additional cases, identify transmission sites, prioritize contacts for screening, and implement control measures (*2*). To understand circumstances of TB transmission that tax local resources, we present an overview of US TB outbreaks during 2002–2008 for which CDC assisted in the investigation. We identified the outbreak population, outbreak contributing factors, the most common transmission sites, and interventions used to control these challenging outbreaks.

## Methods

### Inclusion Criteria and Data Sources

We conducted a retrospective review of TB outbreak investigations in the United States for which CDC provided onsite assistance during 2002–2008. Included in the review were outbreaks having documented evidence of *Mycobacterium tuberculosis* transmission with >3 culture-confirmed TB cases linked by both genotype and epidemiology. Genotyping methods included spoligotyping (all years) and either restriction fragment length polymorphism (2002) or 12-locus mycobacterial interspersed repetitive units (MIRU) (2003–2008). Linkage by epidemiology meant known exposure to another outbreak patient by sharing enclosed airspace in the same period. Linkage by genotype required matching results by whichever genotyping methods were used for that outbreak (generally by spoligotype and either restriction fragment length polymorphism or 12-locus MIRU).

We systematically abstracted data on case characteristics, outbreak contributing factors, transmission sites, and interventions (including contact investigation) from reports written by CDC teams upon return from outbreak investigations. All reports were uniformly written (with background, methods, results, discussion, and conclusion sections) and included aggregate data on demographic, clinical, and social risk factors of cases, epidemiologic linkages, genotyping results, and contact investigations. These data covered the period of the onsite investigation and were either provided by the health departments or collected by CDC as part of the investigation. The reports also discussed the cause of the outbreak and were sent back to the respective local and state health departments with recommendations to further assist in achieving control of the outbreak. We referred to any subsequent presentations and publications describing the investigation (*3**–**19*) to check data quality and accuracy of outbreak contributing factors and transmission sites. For any discrepancy in number of outbreak cases, we deferred to the CDC reports to ensure consistency in the period of data collection across all outbreaks included in the review.

### Definitions

We defined outbreaks based on CDC guidelines for contact investigation as detection of TB disease among >2 persons exposed to a person with infectious TB (*20*), i.e., >3 linked cases within 2 years*.* The standard National TB Surveillance System variables were abstracted for all patients (*21*). Demographic, medical, and social characteristics were ascribed to an outbreak if >50% of the patients involved in the outbreak had that particular characteristic. For outbreak contributing factors, hotspots, and interventions, discrete categories were created based on recurring themes in the reports to enable systematic abstraction and quantification of these variables. Because multiple outbreak contributing factors and a large number of interventions were documented in CDC reports for each outbreak, we abstracted 2 factors considered by the onsite investigators to be key to fueling the outbreak and 3 interventions not already being used that were either used by the CDC team onsite or recommended to be pivotal to the overall control effort. The frequency that each category was encountered was totaled to quantify these outbreak variables. Whenever feasible, a member of the original team investigating the outbreak was consulted to review the accuracy of abstracted data.

Prolonged infectious period was defined as >3 months between symptom onset and the date that effective treatment had been administered for 2 weeks (*20*). Delayed diagnosis was defined as >2 months between symptom onset and date that the patient first sought care for TB symptoms or that TB treatment was initiated (*22**,**23*). Incomplete contact investigations meant inability to either locate or complete evaluation of contacts because of limited resources or a hard-to-reach population. Hotspots were defined as transmission sites where >2 outbreak patients had spent substantial time together, as determined by local public health investigators. Drug house was defined as a venue characterized by the sale or use of illicit drugs. Household was defined as a residential location without documented illicit drug use.

## Results

Of the 51 TB investigations in which CDC participated during 2002–2008, a total of 27 met the inclusion criteria. Twenty-four were excluded for the following reasons: 12 investigations (2 included patients with organ transplants) involved <3 cases; 5 had <3 cases linked by genotype and epidemiology; 5 were international investigations; and 2 had insufficient data in CDC reports.

### Patient Summaries

The 27 outbreaks included in the review involved 398 patients (median 10 patients/outbreak, range 3–35 patients). Of these patients, 364 (91%) were US-born and 50 (13%) were <15 years of age (Table 1). Three hundred thirty-three (84%) had pulmonary disease, including 204 (61%) and 284 (85%) with smear-positive and culture-positive TB, respectively. Eighty-nine percent of isolates (253 of 284) were susceptible to first-line TB medications (Table 1). Of the 197 patients for whom the reason for initial TB evaluation was documented, 74 (38%) were evaluated because of TB symptoms, and 57 (30%) were detected as part of a contact investigation. Ninety-nine (25%) patients required hospitalization, and 23 (6%) died. Infectious periods were documented for 172 patients; the mean and median infectious periods were 6 and 5 months, respectively (range 1–56 months).

**Table 1:** Characteristics of patients in CDC–investigated TB outbreaks, United States, 2002–2008

Characteristic	No. (%) patients
Total	398 (100)
Demographics	
US-born	364 (91)
Black	265 (67)
Male sex	259 (65)
White	66 (17)
Age <15 y	50 (13)
Hispanic	31 (8)
Clinical signs and outcomes	
Pulmonary TB	333 (84)
Cavity on chest radiograph	122 (37)
Sputum acid-fast bacilli smear positive	204 (61)
Sputum culture positive	284 (85)†
Susceptible to first-line tuberculosis medications	253 (89)
Hospitalization	99 (25)
Death	23 (6)

*TB, tuberculosis; CDC, Centers for Disease Control and Prevention. †An additional 10 patients had non-sputum specimens that were culture-positive.

Most patients did not have established medical risk factors for TB (Table 2). Although the total number of patients tested for HIV was not available, HIV infection was documented for 45 patients (12%). Multiple social risk factors for TB were documented: 233 (58%) patients reported alcohol abuse or use of illicit drugs, 126 (32%) had a history of incarceration, and 78 (20%) had a history of homelessness. Sixteen (4%) patients were documented to have a previous diagnosis of TB; of these, 7 (44%) had received incomplete TB treatment.

**Table 2:** Tuberculosis risk factors for patients in CDC–investigated TB outbreaks, United States, 2002–2008*

Risk factor†	No. (%) patients
Total	398 (100)
Medical	
HIV co-infection	46 (12)‡
Diabetes	23 (6)
Immunosuppression (not HIV associated)	14 (4)
History of TB	16 (4)
Incomplete treatment	7 (44)
Social	
Any substance abuse	233 (58)
Alcohol abuse	204 (51)
Nonintravenous drug use	117 (29)
Intravenous drug use	19 (5)
Incarceration history§	126 (32)
Homelessness	78 (20)

*TB, tuberculosis; CDC, Centers for Disease Control and Prevention. †As documented in CDC reports of onsite investigation with information generally gathered through patient chart reviews or interviews. ‡Minimum estimate because complete data on the number of patients tested were not available. §Time frame before TB diagnosis not always documented in CDC reports. The National Tuberculosis Surveillance System collects data on incarceration at time of TB diagnosis.

Spoligotype and MIRU genotype data were available for 22 of 27 outbreaks. Three outbreaks involved a Beijing strain (spoligotype 000000000003771, MIRU 223325173533); the remaining 19 had genotypes that differed from each other. The most frequent strain lineage in these outbreaks was EuroAmerican.

### Outbreak Summaries

US birth and substance abuse were predominant features of outbreaks (Table 3). On the basis of >50% of outbreak patients having a particular characteristic, the criterion used to ascribe a characteristic to an outbreak, 24 of the total 27 outbreaks were characterized by US-born persons, and 18 outbreaks by patients with reported substance abuse. Fourteen (52%) outbreaks were characterized by US-born men who used alcohol to excess or illicit drugs, i.e., marijuana, cocaine, methamphetamine, or heroin. All 8 outbreaks characterized by patients with incarceration histories and the 4 characterized by homelessness had a predominance of patients with reported substance abuse.

**Table 3:** Predominant characteristics of CDC-investigated TB outbreaks, United States, 2002–2008*

Characteristic	No. (%) outbreaks
Total	27 (100)
US born	24 (89)
Male sex	22 (81)
Substance abuse (alcohol/drugs)	18 (67)
Acid-fast bacilli smear positive	17 (63)
Non-Hispanic black	16 (59)
Incarceration history	8 (30)
Cavitary disease on chest radiograph	7 (26)
Non-Hispanic white	4 (15)
Homelessness	4 (15)
Hispanic	3 (11)
HIV co-infection	1 (4)

*Each outbreak had >50% of patients with the select characteristic. TB, tuberculosis; CDC, Centers for Disease Control and Prevention.

Of the 24 outbreaks that occurred among predominantly US-born persons, 21 outbreaks involved substance abuse. For 17 (71%) outbreaks, >50% of patients reported substance abuse; for 2 additional outbreaks, >40% of patients reported substance abuse; and in 2 others, the source patients who reported substance abuse had prolonged infectious periods during which TB was transmitted. In all, 21 (87%) of 24 outbreaks of predominantly US-born patients could thus be characterized as being related to substance abuse. The remaining 3 outbreaks associated with US-born persons were characterized by delayed diagnosis that resulted in transmission in a health care facility (*6*), among family members of a recently incarcerated patient (*7*), and among family members of an undocumented worker (*17*).

Three of the 27 outbreaks occurred among predominantly foreign-born persons. In 1 of these outbreaks, all patients engaged in substance abuse, and although these patients were foreign-born, they had been in the United States for more than a decade (*18*). The other 2 outbreaks among foreign-born persons involved transmission in school, church, and household settings. In each outbreak, the foreign-born patients did not access health care (caused by, in 1 outbreak, fear of repercussions for being undocumented, resulting in multidrug-resistant TB transmission among family members [*17*]).

### Outbreak Contributing Factors and Hotspots

Table 4 describes the most common outbreak contributing factors, which for 24 outbreaks was prolonged infectiousness. In 4 outbreaks in which patients delayed seeking medical attention for their TB symptoms, in 7 where provider-related diagnostic delays occurred, and in 1 where both types of delay occurred, >40% of patients had reported substance abuse. Incomplete contact investigations because of limited resources or a hard-to-reach population contributed to 10 outbreaks.

**Table 4:** Factors contributing to 27 CDC–investigated TB outbreaks, United States, 2002–2008*

Factor	No. outbreaks†
Prolonged infectious period	24
Provider related	
Delayed diagnosis	12
Inappropriate treatment	2
Patient related	
Delayed diagnosis because of late access to care	6
Nonadherence with treatment	5
Mistrust or fear of public health system	6
Incomplete contact investigation	10
Crowded setting with high-risk population	7

*TB, tuberculosis; CDC, Centers for Disease Control and Prevention. †Categories not mutually exclusive.

Drug house was the most commonly identified hotspot (17 outbreaks), followed by homeless shelter (n = 5), correctional facility (n = 4), household (n = 4), workplace (n = 4), church (n = 3), bar (n = 2), school (n = 1), and automobile (n = 1). Ten of the 17 drug houses were primarily residences with extended family members, and the other 7 were largely venues where unrelated persons gathered strictly for the use of illicit substances. In the 4 household outbreaks, crowded living conditions among extended families (primarily foreign-born in 2 outbreaks) were the main TB risk factors.

### Interventions

The most frequently documented intervention to control the outbreak was to prioritize contacts based on risk for infection and progression to disease to ensure that the highest risk contacts were completely evaluated (14 outbreaks). This intervention was necessitated by the large number of contacts identified; contact investigation of 398 patients had generated 16,559 contacts. Of these contacts, 10,142 (61%) had been evaluated by the time of the onsite investigation; 2,128 (21%) were found to have latent TB infection (range 4%–65% per outbreak). Other frequently used interventions included educating community health care providers, e.g., emergency departments, to be vigilant for TB in patients seeking treatment at their facilities (13 outbreaks), and location-based screening, which involves offering TB screening to potential contacts at that particular outbreak’s hotspot or other convenient location (10 outbreaks).

## Discussion

US-born persons who reported substance abuse most strongly characterized the TB outbreaks in this review: 24 of the 27 total outbreaks involved primarily US-born patients, and 19 of these outbreaks involved >40% of patients with reported drug or alcohol abuse. This predominance of substance abuse suggests that it remains one of the greatest challenges to controlling TB transmission in the United States.

Because this descriptive review of TB outbreaks in the United States was restricted to investigations that prompted public health jurisdictions to request CDC assistance, it might lack generalizability to all TB outbreaks in the United States. Outbreaks involving hard-to-reach populations, such as those involving substances abuse or homelessness, with a tendency to overwhelm local public health resources, might be overrepresented. On the other hand, social risk factors such as substance abuse that are based on self-reported behavior might have been underdisclosed because of associated social stigma. Data on key medical risk factors such as HIV and diabetes might have been pending or missing during an investigation and therefore not systematically included. Because genotyping might not have been conducted on every culture-positive case in the jurisdictions affected by these outbreaks, especially during the first 2 years of this review, some cases could have been missed, underestimating the scope of these outbreaks. Despite these limitations, characteristics found to be associated with intense TB transmission are consistent with findings in the previous literature.

Although the case rate is 10× higher among foreign-born than among US-born persons (*21*), this disparity was markedly lacking in our review; 91% of outbreak patients were US born. Prior studies have demonstrated that recent transmission occurs mainly among US-born persons, with foreign-born persons more likely to develop reactivation of latent TB infection acquired before immigration (*24**–**26*). Similar to other studies (*25**–**27*), our few examples of TB outbreaks among immigrants were all associated with crowded living conditions and lack of access to medical care, whereas outbreaks that involved mainly US-born persons were associated with substance abuse and other risk factors, such as homelessness and incarceration.

Among nationally reported TB cases, substance abuse has been estimated to be the most prevalent modifiable TB risk factor, reported by 29% of US-born vs. 8.3% of foreign-born patients (*28*). In our overview, 58% of outbreak patients self-reported substance abuse. Consistent with national TB surveillance regarding substance abuse, alcohol was the most commonly reported substance. Alcohol has been documented to increase the risk for TB exposure, susceptibility to infection, and progression to active disease (*29**,**30*). Contact investigations among bar patrons have yielded latent TB infection rates of 40%–50% (*29*,*31*), highlighting the transmission risks in this population. Failure by contacts who abuse alcohol to be treated for latent TB infection can prolong outbreaks if active TB subsequently develops in these persons and they then serve as additional sources of transmission (*4*).

Substance abuse is a long-established risk factor for TB infection and disease (*32**,**33*), but in recent years its role in fueling TB transmission has also been recognized (*10**–**12**,**14**,**15**,**18**,**19**,**28**,**29**,**31**,**32*). Persons who report substance abuse are associated with increased TB transmission because of sociobehavioral and clinical TB risk factors. First, persons who report substance abuse are more likely to have smear-positive disease and experience treatment failure (*28**,**34*), e.g, because of nonadherence, both of which can increase infectiousness (*20*). The higher prevalence of smear positivity might be attributed to delayed diagnosis, or, in cases of crack cocaine use, pulmonary damage that leads to alveolar macrophage impairment and cytokine dysfunction (*34*). Second, persons who report substance abuse are likely to experience a prolonged infectious period because of delays in seeking medical care and, once they are medically evaluated, receiving a TB diagnosis (*18**,**31**,**32*). Third, sharing of drugs or alcohol often occurs in confined and poorly ventilated settings such as drug houses (*4**,**10**–**12**,**15**,**18**,**19*), bars (*15**,**29**,**31*), homes (*7**,**10*), and vehicles (*18*)—all of which facilitate close and prolonged contact. The most common hotspots in this review were settings in which drug use occurred; poverty, unstable housing, and overcrowded conditions exacerbated TB transmission (*4**,**7**–**9**,**11**,**15**,**18**,**19*). Fourth, contacts of TB patients are often difficult to identify because patients want to protect the names of contacts with whom they engage in illicit or other activities perceived to have social stigma (*10**,**28**,**31**,**32*). Our finding of an overall 21% latent TB infection rate among contacts, lower than the expected 30% (*20*), might reflect evaluation of relatively lower risk contacts whose names were easier to elicit. Finally, contacts with substance abuse can be difficult to locate, be less likely to accept and adhere to treatment, and have a greater risk for adverse reactions from medication, e.g., related to interaction of alcohol with isoniazid (*28**,**29**,**32*).

Given the predominance of patients with substance abuse in our review, it is not surprising that prolonged infectious period was the most common outbreak contributing factor. Delayed diagnosis was the most common cause (14/27 outbreaks) and has been cited as a major contributor to TB outbreaks (*3**,**7**,**11**–**15**,**22**,**23**,**25**,**27**,**35**,**36*). In 1 outbreak, during a 1-year infectious period, the source patient lived in 4 locations, all crowded settings, and shared illicit drugs with household members, facilitating TB transmission to 3 adults and 3 children (*11*). In another outbreak, during the 9 months that the source patient’s diagnosis was delayed, the patient was in and out of jail and had multiple moves to new residences, resulting in 37 additional cases (including 10 children) across 3 counties (*9*). These examples of intense transmission occurring before a correct diagnosis was made highlight the need for educating health care providers to suspect TB when encountering either persons born abroad or domestically with social risk factors for TB, such as substance abuse, homelessness, and incarceration history (*6**,**7**,**9**,**11**,**12**,**15**,**35*). Failure to do so can lead to outbreaks that overwhelm public health resources*.* Additionally, raising general awareness about TB so that patients seek early medical care and know the value of completing treatment are critical to ending transmission (*22**,**36*).

This review found that incomplete contact investigation was the second most common contributing factor to TB outbreaks. When contact investigations are incomplete, a pool of latent TB infection remains, threatening to generate additional cases and cause ongoing transmission (*10**,**15**,**24*). Compounding these risks, persons who report substance abuse are more likely to be poor, homeless, and have an incarceration history—all documented TB risk factors (*5**,**11**,**13**–**15**,**28**,**37**,**38*). When contact investigations involve a hard-to-reach population, conventional methods of contact tracing may need to be expanded to include other approaches (*2**,**5**,**10**,**13**–**15**,**27**,**39*). To optimize the yield of contact investigation, the 2 interventions most frequently used in these outbreaks were prioritizing screening of contacts on the basis of TB risk (*3**,**5**,**6**,**8**–**11**,**18*) and offering location-based TB screening at specific venues associated with each outbreak, including bars, shelters, and drug houses (*5**,**6**,**10**,**13**,**14*). Although this intervention is resource-intensive, its benefits have been recognized in several investigations involving hard-to-reach populations (*10**,**14**,**31*). In 1 outbreak, unnamed contacts encountered at a drug house frequented by numerous TB patients were offered screening and were found to be 8× more likely to have a positive tuberculin skin test result than were named contacts (*10*).

Although this review was limited to outbreaks in which CDC was invited to assist and might not represent all TB outbreaks in the United States, it provides an opportunity to identify common themes among outbreaks which, when present, tend to challenge local public health capacity. These outbreaks featured US birth and substance abuse—factors shown to be independently associated with genotype clustering, a marker for recent TB transmission (*39**,**40*). Although TB incidence has been decreasing in the United States, its elimination will not be achieved without more effective strategies to prevent, detect, and treat TB among persons who are known to abuse substances.
